# New Explosive-Circulation Technology of Tire Recycling for the Production of Crumb Rubber with Modified Surface

**DOI:** 10.3390/polym17091260

**Published:** 2025-05-05

**Authors:** Vyacheslav M. Misin, Alexander A. Nabok, Alexander A. Zakharov, Alexey V. Krivandin, Natalia I. Krikunova, Vladimir A. Volkov, Mikhail V. Voronkov, Sergey I. Pozin, Alexey K. Buryak, Alexander E. Tarasov, Alexander V. Naumkin, Sergey S. Nikulin

**Affiliations:** 1 N. M. Emanuel Institute of Biochemical Physics of the Russian Academy of Sciences, 4, Kosygin St., Moscow 119334, Russia; misinnn@gmail.com (V.M.M.); a.krivandin@sky.chph.ras.ru (A.V.K.); kric@mail.ru (N.I.K.); voronkov330363@yandex.ru (M.V.V.); 2 Explotex Group LLC, 6, 1st Dorozhny Proezd, Moscow 117545, Russia; nabok.1@yandex.ru (A.A.N.); explotex1@mail.ru (A.A.Z.); 3 A. N. Frumkin Institute of Physical Chemistry and Electrochemistry, 31, Leninsky Prospect, Moscow 119071, Russia; sipozin@yandex.com (S.I.P.); akburyak@mail.ru (A.K.B.); 4 Federal Research Center of Problems of Chemical Physics and Medicinal Chemistry RAS, 1, Academician Semenov Ave., Chernogolovka, g.o. Chernogolovka, Moscow 142432, Russia; atarasov@icp.ac.ru; 5 A. N. Nesmeyanov Institute of Organoelement Compounds, Russian Academy of Sciences, 28/1, Vavilov Street, Moscow 119334, Russia; naumkin@ineos.ac.ru; 6 Federal State Budget Educational Institution of Higher Education Voronezh State University of Engineering Technologies, 19, Revolution Avenue, Voronezh 394036, Russia; nikulin.nikuli@yandex.ru

**Keywords:** waste tires, explosion-circulation technology, plant, crumb rubber, nanoglobules

## Abstract

The article reports on the development of a fundamentally new, effective technology for recycling waste tires using the explosive-circulation technology method, which was implemented in industry at a working factory. The construction of an explosion-circulation reactor, in which tires are destroyed under the influence of an explosion, is described. The main technological stages of the reactor operation include the formation of a tire package with a height of about 2.4 m and a mass of up to 1000 kg; cooling the package by air turbo-cooling machine to a temperature of minus 70–80 °C; placing the package into the reactor; initiating the explosive charge; and removing the tire shedding products with a subsequent granulometric classification of the resulting rubber crumb. The resulting rubber crumb has good wettability, which eliminates the need for an additional technological stage of activating the crumb surface. This made it possible to successfully use the obtained rubber crumb to improve the characteristics of road construction bitumen, the hardness of which at −16 °C decreased from 217 to 161 MPa. Using atomic force microscopy (AFM), gas chromatography, mass spectrometry, GPC, and XPS, it was established that the good wettability of the crumbs is explained by the formation of molecules with polar groups (C-O, C=O, C(O)O, C-S, C-SO_x_, Zn-S, O-Si(O)-O) on the crumb surface as a result of the explosion.

## 1. Introduction

Car sales are increasing worldwide. In parallel with this, the number of purchased tires increases and, therefore, the number of worn tires, which represent the largest volume of polymer-containing waste that is practically not subject to decomposition. Therefore, the problem of recycling used car tires is of great environmental and economic importance for all developed countries in the world. According to various estimates, more than 1.6 billion new tires are produced worldwide every year, and 1–1.5 billion used tires are discarded, which has an extremely negative impact on the environmental situation, especially considering the long-term consequences. In the US alone, there are at least 275 million used tires in warehouses [1], with annual growth projected at 4%, and global tire sales reaching USD 268 billion annually [2].

Various technologies are used for tire recycling. The resulting waste tire processing products have their own commercial value. Taking into account environmental requirements, investments in tire recycling technologies are economically feasible [3]. The global tire recycling market will reach USD 7.32 billion by 2031, driven by processing technologies (pyrolysis and shredding), products (rubber mulch, rubber powder, fillers, fuel), and end users [4].

The developed tire recycling technologies are proposed to be classified using various criteria, and several areas of tire recycling can be distinguished:

- Physical destruction of tires, including cryodestruction and ozone knife methods, allowing to obtain pieces of rubber, shredded rubber, and crumb rubber suitable for creating any materials [5,6,7,8,9,10,11,12];
- Barodestruction of tires, in which tire rubber is brought to a fluid state in an industrial press;
- Pyrolysis. This tire recycling method produces pyrolysis oil, carbon residue, and metal cord. Pyrolysis oil can be further purified and used as fuel. Carbon residue can be used to make new rubber or dyes. Metal cord is remelted [13];
- Foam concentrates in electric arc furnaces [14];
- The use of tires as energy sources, including in cement kilns [15,16,17].

Unfortunately, some technologies have their own disadvantages, including harmful emissions, poor product quality, and low profitability. The analysis of available data indicates that the most used recycling product is crumb rubber [9]. For this reason, known technologies for processing tires into rubber crumb are being optimized, and new ones are being developed. Depending on the size of the crumb, it is recommended to use it in rubberized cement–concrete mixtures [2,12,18,19,20,21,22,23,24,25,26,27,28,29], in bitumen and asphalt mixtures [1,11,23,29,30,31,32,33,34,35,36,37,38,39], in polymer concrete based on epoxy resin [5,40,41,42,43,44], in polymer composites [7,10,12,45,46,47,48], and as a sorbent [49].

On the surface of rubber crumb fractions obtained using the mechanical technologies described above, nanoglobule formation is never observed, which reduces the crumb’s reactivity in forming bonds with the host material. Additionally, the product lacks wettability, making it unsuitable for use in concrete or water-based mixtures due to cavity formation. An important research direction is the development of additional technologies for activating the surface of rubber crumb [5,11,32,38,39,50,51,52]. The modification of the rubber crumb surface improves its adhesive properties, which naturally increases the quality of composites from this crumb. However, additional surface modification processes lead to the emergence of additional technological stages and to an increase in the cost of the final product. Thus, the literature analysis shows the need to develop and use technologies for the production of rubber crumb with an activated surface. This article reports on a new effective method for recycling worn-out car tires using a new, original patented technology of explosive circulation [53,54] and the properties of the resulting regenerate in the form of rubber crumb. It should be emphasized that the modification of the rubber crumb surface occurs already at the main stage of the tire recycling process without the use of additional stages of activation of the surface of the resulting crumb.

The described process has now been implemented in industry by Explotex. Moreover, the successful implementation of this technology has become the basis for designing a reactor capable of processing large-size tires. An explosive-circulation technology for recycling heavy-duty tires from quarry trucks is currently being designed. The article describes the main features of the new tire recycling technology, which ensures good wettability of the resulting rubber crumb. The reasons for good wettability are explained using various physical and chemical research methods.

## 2. Materials and Methods

### 2.1. Materials

The following tire types were subjected to explosive-circulation grinding for the research:

1. Radial solid-steel cord: 385/65R22.5; 315/60R22.5; 295/80R22.5; 245/70R; 215/75 R (Michelin, Bridgestone, Continental, as well as those made in Russia, a total of about 38,000 kg);
2. Radial solid-steel cord: 385/65R22.5; 315/60R22.5; 295/80R22.5; 215/75 R (People’s Republic of China, a total of about 6000 kg);
3. Diagonal tires: 3270 kg;
4. Tires with the maximum possible visible defects on the surface associated with long-term storage (about 1000 kg);
5. Aircraft tires with textile cord (about 1000 kg).

A typical tire production uses rubber, metal cords, and textile cords.

### 2.2. Methods

#### 2.2.1. Wettability Test

When testing the crumb for wettability, weighed samples of rubber crumb were placed in containers with water. Mixing was carried out for 5 min (30 min for CR 0–1). After mixing, some of the rubber crumb sank to the bottom of the container. The rest was stable on the surface of the water. All rubber crumb (floating on the surface and located at the bottom of the container) was removed, dried, and weighed. The total mass of the floating and sinking crumbs was equal to the original mass of the crumbs.

#### 2.2.2. X-Ray Diffraction Study

The structure of rubber crumbs was studied by X-ray diffraction in the region of wide- and small-scattering angles. The wide-angle X-ray diffraction patterns were obtained on an HZG4 X-ray diffractometer (Freiberger Präzisionsmechanik, Freiberg, Germany) according to the Bragg–Brentano X-ray optical scheme (Cu X-ray tube, 40 kV, 20 mA, Ni-filter, 5–60° 2θ interval, 0.05° 2θ step size). The intensity of small-angle X-ray scattering was measured using an X-ray diffractometer with a position-sensitive detector (Cu X-ray tube, 30 kV, 20 mA, Ni-filter, 340 mm sample to detector distance, 0.096 mm detector channel size) in the range of diffraction vector modulus values of 0.025 nm^−1^ < S < 1.6 nm^−1^ (S = (2sinθ)/λ, 2θ is the scattering angle and λ is the wavelength of X-ray radiation equal to 0.1542 nm for CuKα). Experimental small-angle X-ray scattering curves were corrected for background scattering and desmeared as in [55].

#### 2.2.3. Atomic Force Microscopy Study

The topography of the regenerated rubber crumb surfaces was carried out in semi-contact mode on a Veeco probe microscope (MultiMode model with Nanoscope IV controller (Veeco, Plainview, NY, USA) using NT-MDT NSG-10 probes (NT-MDT, Russia, Zelenograd) (radius of curvature 10 nm, hardness coefficient 5.5–22.5 N/m, resonance frequency 190–325 kHz).

#### 2.2.4. Gel Permeation Chromatography Study

The GPC analysis of the samples was performed using a Waters GPCV 2000 chromatograph (Waters corporation, Milford, MA, USA) with a refractometric detector. Styrogel columns of the PLgel 5 μm MIXED-C (Agilent Technologies Inc., Santa Clara, CA, USA) brand with a length of 300 mm and a diameter of 7.6 mm were used at a temperature of 35 °C. Tetrahydrofuran was used as an eluent with an elution rate of 1.0 mL/min. Molar masses were calculated using polystyrene calibration. The analyzed samples were prepared by extracting from the surface of regenerated rubber crumb with chloroform for 1 h in a Soxhlet apparatus.

#### 2.2.5. Gas Chromatography Study

The analysis of the composition of the gas phase above the surface of the regenerate rubber crumbs was carried out on a Crystal 2000M chromatograph (Chromatek, Yoshkar Ola, Russia) with a flame ionization detector and a quartz capillary column DB-1 (50 ’ 0.32 mm^2^, phase layer 0.25 μm, J&W Scientific, Folsom, CA, USA). The column was programmed to change the temperature from 50 °C (held for 5 min) to 250 °C at a rate of 8 °C/min; the temperature of the injector and detector was 250 °C. The carrier gas (helium) flow rate through the column was 2.5 mL/min, and the column split ratio was 1:30. Volatile substances from the gas phase were isolated and extracted by solid-phase microextraction (SPME) using special equipment (Supelco, Bellefonte, PA, USA).

#### 2.2.6. Thermodesorption Mass Spectrometry Study

Thermodesorption mass spectrometric studies were carried out on a JMS-D300 device (Jeol, Tokyo, Japan) with a JMA-2000 computer (Jeol, Tokyo, Japan) and a proprietary attachment for direct sample input, ensuring research in the temperature range of −30 °C to 400 °C. The thermal desorption was investigated using a direct input in the temperature range of 30–400 °C, and the heating rate of the sample was varied from 2 to 50 °C/min. Ionization with electrons with an energy of 70 eV was used. The recording rate of the spectra in the range of mass numbers 10–300 or 40–450 m/z was varied from 2 to 10 s in order to obtain information about various desorbable compounds and facilitate their identification using library search programs in which literary spectra are presented for different ranges of mass numbers.

#### 2.2.7. X-Ray Photoelectron Spectroscopy Study

The X-ray photoelectron spectra (XPS) were acquired on a Quantera SXM spectrometer (Physical Electronics, Chanhassen, MN, USA) using monochromatized Al Kα radiation at an operating power of 25 W of the X-ray tube. Survey, high-resolution, and C KVV spectra were recorded at pass energies of 280, 50, and 140 eV, with step sizes of 1, 0.1, and 0.2 eV, respectively. Granules were placed on a sample holder using a double-sided nonconductive adhesive tape and analyzed at room temperature at a base pressure lower than 10^−8^ Torr. The base pressure in the analytical UHV chamber of the spectrometer during measurements did not exceed 10^−8^ Torr. The energy scale of the spectrometer was calibrated using the binding energies of the peaks for the Au 4f_7/2_ (83.96 eV), Ag 3d_5/2_ (368.21 eV), and Cu 2p_3/2_ (932.62 eV) core levels of reference samples. The electrostatic charging effects were compensated by using the dual beam neutralizer. After the subtraction of the inelastic background by the Shirley method, the spectra were referenced to the C–C/C–H state identified in the C 1s spectrum, to which a binding energy of 285.0 eV was assigned. The surface chemical composition was calculated using the MultiPak data reduction software (V.8.2).

## 3. Results and Discussion

### 3.1. Characteristics of Explosive-Circulation Technology

**Figure 1:**
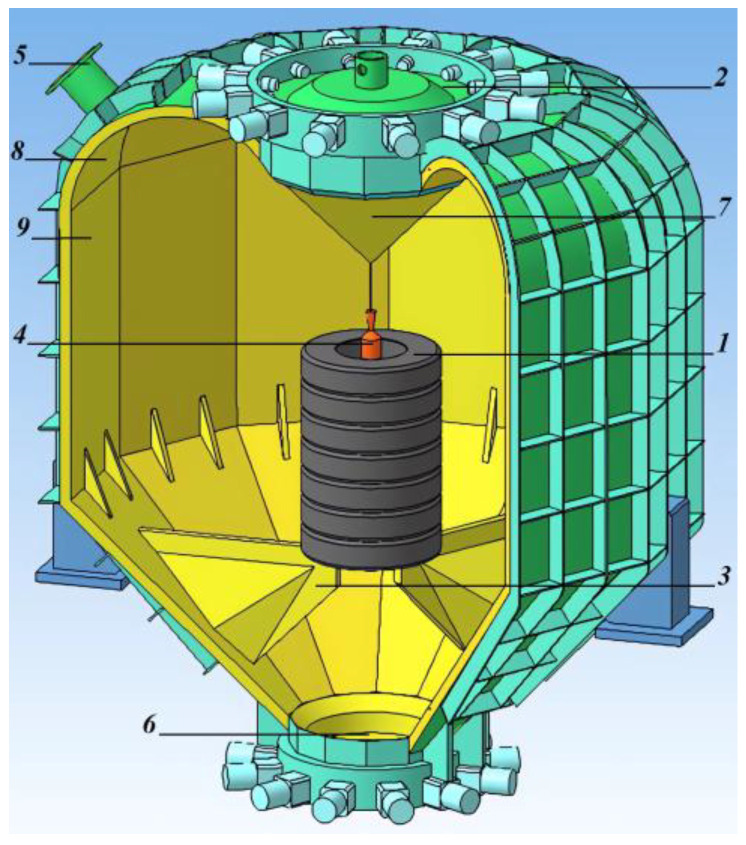
Scheme of the EXPLOTEX-30 explosion circulator. 1—tire package; 2—tire package loading hatch; 3—supports for the tire package; 4—explosive charge; 5—pipe for removing gaseous products; 6—regenerate crumb unloading hatch; 7—fairing cone; 8—internal chute; 9—shell of the working chamber of the explosion circulator.

This section provides a description of the EXPLOTEX-30 explosion circulator (Figure 1), and also describes the main characteristics of the explosion-circulation technology, which was practically implemented at the operating plant.

#### 3.1.1. Description of the Design of the Explosion Circulator and Characteristics of Its Operation

The explosion circulator (Figure 1) is designed to solve the following problems:

- Increase the efficiency of tire shredding by explosion;
- Ensure localization of the explosion and guaranteed protection of personnel and equipment from all types of damaging effects of a charge explosion (shock wave, gaseous products, spread of tire fragments).

The safety of the explosion circulator is ensured by the following:

- The strength (to explosion) of the device is calculated in accordance with the industry calculation method OST-92, which guarantees the operability of the device with a 20-fold safety margin for the destruction conditions;
- The device has passed preliminary strength tests by the action of an explosion of an explosive charge (EC) and has shown a 20-fold safety margin for the destruction conditions.
- Commissioning of the device is carried out in accordance with the permission of Federal Environmental, Industrial and Nuclear Supervision Service of Russia upon fulfillment of a number of requirements that ensure the safe operation of the device (Operating Manual, Blasting Operations Project, Expertise on Exploding Operations Project, etc.).

**Figure 2:**
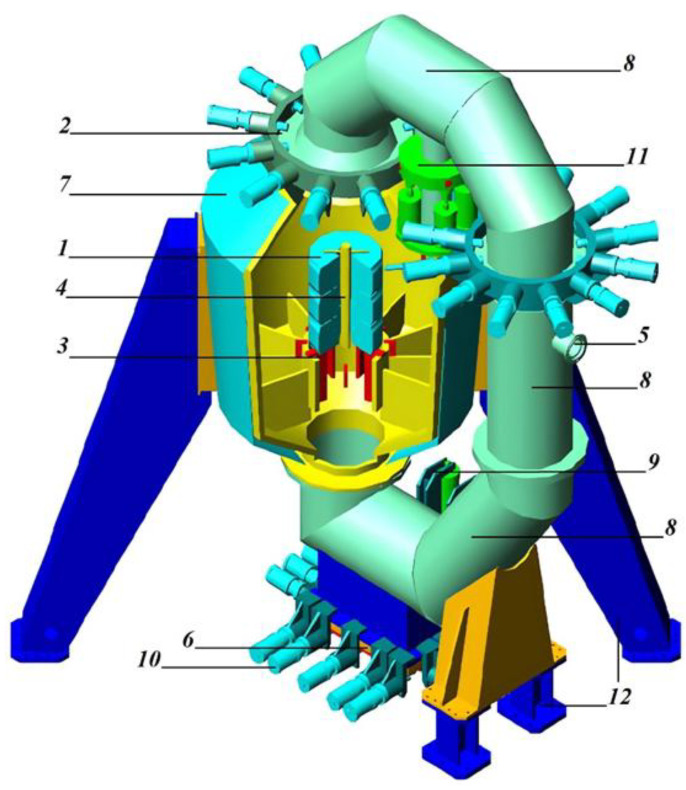
Scheme of the EXPLOTEX-30 explosion circulator, mounted with a closed-ring path through which explosion products and a shock wave circulate. 1—tire package; 2—tire package loading hatch; 3—supports for the tire package; 4—explosive charge; 5—pipe for removing gaseous products; 6—regenerate crumb unloading hatch; 7—shell of the working chamber of the explosion circulator; 8—closed pipes ring tract; 9—mechanism for opening the unloading hatch; 10—mechanisms for sealing the unloading hatch; 11—mechanisms for lifting and rotating the knee; 12—explosion circulator supports.

In addition, the explosion circulator can be operated in conjunction with a closed ring path, which is a system of interconnected large-diameter pipes connecting the upper and lower parts of the explosion circulator (Figure 2). Explosion products and a shock wave, after the tire package explodes, circulate through the working chamber and a closed path. The upper part of the closed path can be lifted and moved to the side, making it possible to load a package of tires through the upper hatch.

**Figure 3:**
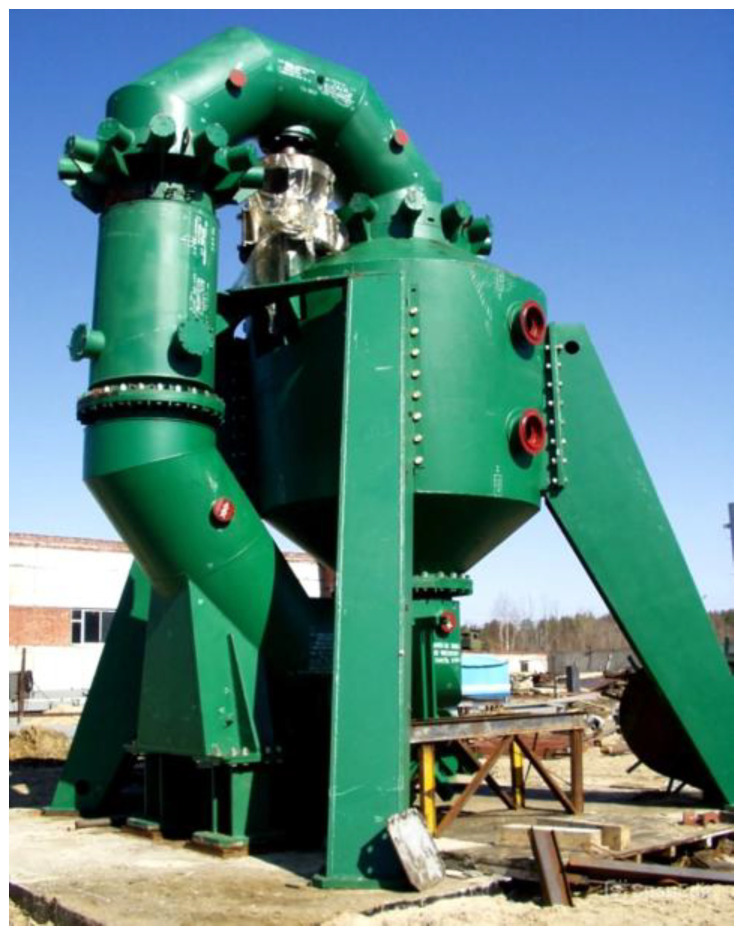
General view of an explosion circulator with a closed annular path.

Figure 3 shows a ready-to-use explosion circulator in combination with a closed ring path.

#### 3.1.2. Description of the Technological Process

Explosive-circulation technology for shredding used tires from cars and trucks consists of the following technological operations:

1. The formation of tire packages with a height of about 2.4 m and a mass of up to 1000 kg (1 in Figure 1). Each package consists of seven tire briquettes stacked on top of each other. Each of the briquettes can contain one truck tire into which three–four car tires or one truck tire is inserted.
2. This package is cooled by air turbo-cooling machines to a temperature of minus 70–80 °C. Then, through the hatch (2 in Figure 1), the package is placed into the explosion circulator and installed on internal supports (3 in Figure 1). A coaxial cylindrical explosive charge is installed inside the tire package (4 in Figure 1). Around the coaxial cylindrical explosive charge there is a cylindrical shell filled with an aqueous solution of technological additives. These additives can significantly reduce the amount of toxic gases generated during an explosion and then escaping through the gaseous products removal pipe (5 in Figure 1).
3. The explosion circulator is sealed. The explosive charge is initiated. Under the influence of gaseous explosion products, the tire package expands with an increase in the diameter of the package by three–four times. This leads to the division of tires into separate factions. The fractions are further crushed upon impact with the surface of the explosion circulator. The explosion circulator is designed in such a way that the shape of its surface causes the movement of shock waves along a trajectory close to a tangent to its surface. After the completion of the circulation of shock waves and explosion products, static pressure of a gaseous medium of up to 1 atm is formed inside the explosion circulator.
4. Gaseous products of the explosion are removed through the pipe branch (5 in Figure 1) into the gas purification system. After the excess pressure of the medium in the explosion circulator becomes equal to zero, the tire-shredding products are removed from the explosion circulator through the hatch (6 in Figure 1).
5. The technology allows for the processing of 10 packages of tires per hour, which equals up to 30 thousand tons of tires per year.

When an explosive charge explodes (4 in Figure 1), the detonation wave moves from the upper end of the charge to the lower. The resulting gaseous products of the explosion and the shock wave first act on the upper briquette of the tire package, causing it to expand in the horizontal direction. Then, as the explosion develops, the remaining briquettes are sequentially loaded by the action of the explosion, each of which expands in the horizontal direction.

The tire package takes the shape of a cone, and the inner part of the package takes the form of a conical horn, which forms the movement of gaseous products and a shock wave in the vertical direction to the top of the fairing cone (7 in Figure 1). The shock wave moves at a small angle to the surface of the cone, then moves along the surface of the chute (8 in Figure 1) and along the surface of the shell (9 in Figure 1) of the explosion circulator. The surfaces of the fairing cone (7 in Figure 1), internal chute (8 in Figure 1), and shell (9 in Figure 1) are smoothly mated to each other, which contributes to the absence of the effect of reflection and increase in pressure in the shock wave. The shock wave begins to circulate in the working chamber of the explosion circulator. It moves in a direction that is close to the direction tangent to the mating surfaces. In this case, the shock wave acts on the surface with its internal pressure without reflection. Gaseous products of the explosion are directed through the pipe (5 in Figure 1) into the gas purification system.

In an explosion circulator, using a closed annular path (Figure 2 and Figure 3), the shock wave is directed into the path, it circulates through the closed path and the working chamber, and moves in a direction tangential to the surfaces of the path and the working chamber. This reduces the load on the explosion circulator. In addition, the products of the destruction of the tire package are involved in the circulation process, which contributes to their additional grinding. However, the use of a circular closed path leads to an increase in the overall height of the explosion circulator. In turn, this leads to the need to increase the height of the production facility.

As a result of the explosion, a crushed product is formed. It is a mechanical mixture with an average composition of the following (100% is taken as the initial mass of the tires being shredded):

1. Rubber crumbs with particle sizes of 0–10 mm—up to 65% of the total mass of crushed tire packages.
2. Metal cord—19–23% in the form of needles and wire tangles, cleared of rubber (with rubber residues up to 1–2%).
3. Textile cord—8–12%, in the form of short threads and fluff.

After the tires have been crushed by the explosion, the steel wire (metal cord) is separated from the rubber fractions by magnetic separation. There is no need to clean the surface of the rubber crumb from the explosion residues. The gaseous products of the explosion are converted into harmless products by means of microparticles of an aqueous solution containing technological additives and sprayed by the action of the explosion. This solution is in a cylindrical shell, which is placed around a coaxial cylindrical charge of explosive (described above). Control is carried out on the basis of sanitary standards.

The resulting rubber particles, separated by industrial sieves, are sent to various conveyors in accordance with the established sizes of the sieve cells and end up in various containers:

- Particles with a size of 0–1 mm (CR 0–1 mm) make up to 30% of the rubber mass;
- Particles with a size 1–3 (CR 1–3 mm), up to 25%;
- Particles with a size 3–5 mm (CR 3–5 mm), up to 20%;
- Particles with a size of 5–10 mm (CR 5–10 mm), up to 20%;
- Particles larger than 10 mm, approximately 10%.

**Figure 4:**
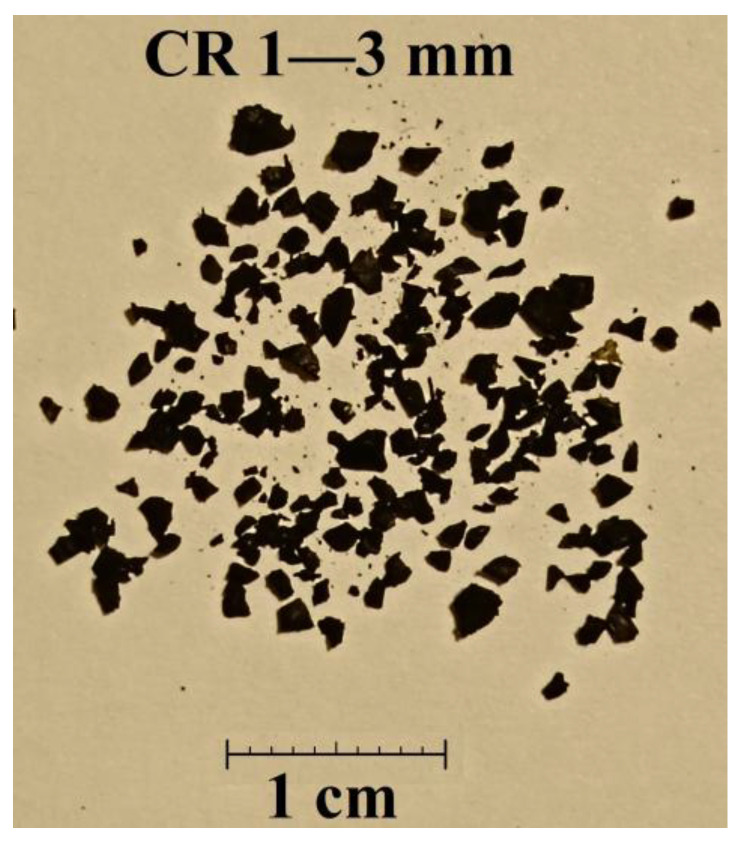
Photographs of the regenerate fraction CR 1–3 mm.

The exact amount of each fraction depends on the type of specific car tires in the package being destroyed. Figure 4 shows, as an example, a photograph of fraction CR 1–3 mm.

#### 3.1.3. Description of the Explosive and Strength Characteristics of the Explosion Circulator

A granulated mixture of ammonium nitrate, aluminum powder, and mineral oil is used as an explosive. The mass of the working explosive charge used for the explosion is 40 kg (approximately 4% by weight of the tire pack weight). This is equivalent to 0.9 × 40 = 36 kg of TNT equivalent.

The explosion circulator operates within the limits of elastic deformations if the number of explosives is 249 kg of TNT equivalent (calculated value). The onset of crack formation, without catastrophic destruction of the hull, occurs with an explosion of ≥600 kg of TNT equivalent (calculated value).

#### 3.1.4. Advantages of Explosion-Circulation Technology

A comparative assessment was made of the explosion-circulation technology and mechanical methods of recycling tires, the reclaim of which is also rubber crumb. The explosion-circulation technology has a number of advantages over mechanical methods of crushing:

- Production areas for explosion-circulation technology are three–four times smaller than for mechanical technology;
- The price of equipment for explosion-circulation technology is approximately 30% less than the price of equipment for mechanical technology;
- The energy consumption for crushing one ton of tires using explosion-circulation technology is about 250 kWh/t, or approximately two times less than when using mechanical technology;
- The high wear of equipment used in mechanical method: repair of shredders and sharpening of knives once a week; replacement of knives on average every 2.5 months after nine sharpenings; shredders wear out after 5–10 years. This requires frequent shutdown of the process, which reduces its economic efficiency;
- The detected presence of polar groups on the surface of the crumb produced using explosion-circulation technology leads to a significant improvement in the adhesion of the crumb;
- Rubber crumb produced using explosion-circulation technology has improved adhesion in composites due to the presence of polar groups on the crumb surface, which in turn eliminates additional costs for modifying the crumb surface.

During the operation of the equipment (explosion localization device), mechanical stresses arise in it in accordance with the calculation confirmed by practical results. However, these stresses are less than the fatigue stresses of the material from which the equipment is made. Therefore, the equipment can operate for a long time, providing the possibility of producing a large number of cycles of tire crushing by explosion. The plant for the production of rubber crumb obtained by the explosion circulation method operated for 12 years without major repairs of the equipment.

#### 3.1.5. Application of Crumb Rubber in Composites

This section presents the main results of a study by the engineering company ProfilConsult GmbH (Dortmund, Germany) of 50/70 road-building bitumen modified with crumb rubber CR 1–3 mm in the amount of 10%. Crumb rubber was gradually added to molten bitumen (160 °C) over 30 min. Mixing was carried out with a paddle mixer at a speed of 200 rpm.

The following studies were carried out:

- Needle penetration at 25 °C according to [56],
- Softening point of ring and ball according to [57],
- Deformation behavior in the dynamic shear rheometer (DSR) according to [58] Tests were carried out in the temperature range of 30–90 °C,
- Behavior at low temperatures—bending beam rheometer (BBR) according to [58],
- Elastic recovery according to [59].

A change in the complex of properties of road-building bitumen modified with Explotex reclaimed rubber crumb was discovered. In particular,

- Needle penetration decreased from 50.3 to 39.3 1/10 mm;
- Hardness at −16 °C decreased from 217 to 161 MPa;
- Complex shear modulus in the rheometer increased from 416,100 to 580,300 and from 15,461 to 39,700 Pa at 30 °C and 50 °C, respectively.

It should be noted that with a higher hardness of the bitumen/rubber crumb composition, no ruts are formed on the road surface, which, firstly, reduces the safety of vehicle traffic, and, secondly, reduces the service life of the road surface.

The final conclusion of ProfilConsult GmbH states the following: “The results of rheological and physical studies clearly show that the modification of road construction bitumen grade 50/70 by adding 10% rubber crumb Explotex leads to a significant increase in operational properties (high and low temperatures).”

### 3.2. Structure and Physicochemical Properties of the Resulting Crumb Rubber

Good compatibility of bitumen with rubber crumb and the corresponding improvement in the performance properties of modified bitumen indicate the specificity of the surface of this crumb obtained by the explosive-circulation method. Therefore, the physicochemical properties were studied for four fractions of crumb rubber CR 0–1, CR 1–3, CR 3–5, and CR 5–10. For comparison, crumb rubber RD-0.8 with a size of 0.3–0.63 mm, obtained by mechanical shredding, was studied.

#### 3.2.1. Wettability of Regenerated Crumb Rubber

The wettability of the regenerate crumb was assessed, since good wettability of the crumb should ensure good compatibility of the crumb with the matrix, and therefore good physical and mechanical properties of the corresponding composites. It was found that placing regenerated rubber crumbs in containers (vessels) with water leads to the sinking of most of the rubber crumbs (84–94%) to the bottom of the vessels for almost all fractions within 5 min. For the CR 0–1 fraction, this value was lower (60% in 30 min). The observation of the surfaces of crumb rubber with an optical microscope revealed the presence in the rubber crumb of finely dispersed fractions of textile fiber (crushed tire cords) that are not wetted by water. It is natural that the largest amount of fiber was found in the CR 0–1 fraction, which has worse wettability. This is explained by the small size of textile fibers, which are separated together with the smallest rubber crumb of fraction CR 0–1 during the technological separation of the regenerate formed after the explosion. The good wettability of the regenerate with water and the effectiveness of its use in compositions containing matrices with polar groups can only be explained by the presence of polar groups on the surface of the crumbs. On the other hand, crumb rubber produced by mechanically shredding tires using roller technology settled to the bottom of the vessels much worse: only 11% in 30 min. This result is natural, since this tire rubber crumb consists of non-polar rubber molecules that are not wetted by polar solvents, including water.

#### 3.2.2. X-Ray Diffraction Study

**Figure 5:**
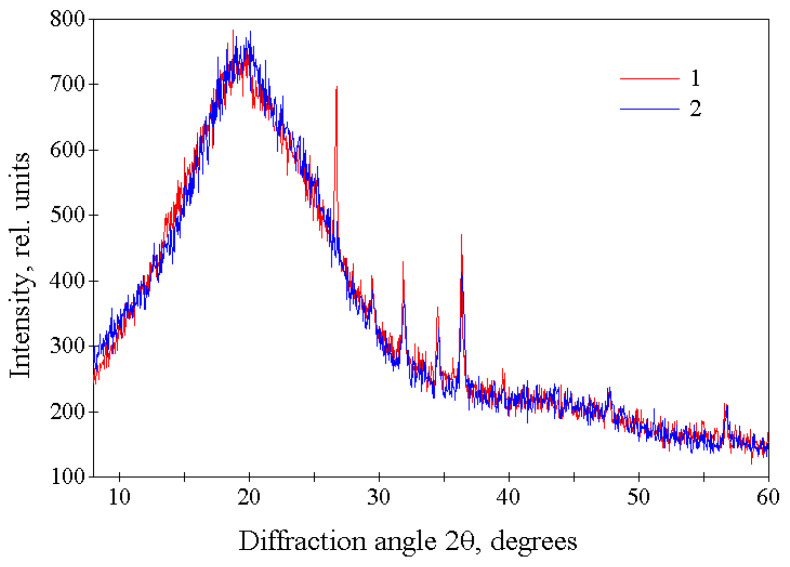
Wide-angle X-ray diffraction patterns of tire material crushed in an explosion-circulation plant (1—CR 0–1) and mechanically (2—RD 0.8).

During the process of explosive-circulation destruction of tires, a restructuring of the supramolecular structure of the destroyed substance could occur. Therefore, to study the possible features of the structure of the crumb rubber obtained by the explosive-circulation method, a comparative X-ray diffraction study was carried out on samples of this crumb and the rubber crumb obtained by mechanical shredding. Diffraction patterns in the region of wide-angle scattering for crumb rubber samples obtained using explosive-circulation technology and traditional mechanical methods are very similar (Figure 5). The main broad diffraction peak in these diffraction patterns, centered at 2θ ≈ 20°, is typical of polymer amorphous structures. In addition, these diffraction patterns contain narrow low-intensity diffraction peaks (2θ ≈ 26.7, 29.5, 31.9, 34.5, 36.4, 39.6, 47.7, and 56.7°). The total integrated intensity of all these peaks does not exceed ~3% of the intensity of the amorphous polymer. These peaks indicate that the tire material contains a small amount of large crystalline inclusions. The identification of these inclusions was not a task of this work.

**Figure 6:**
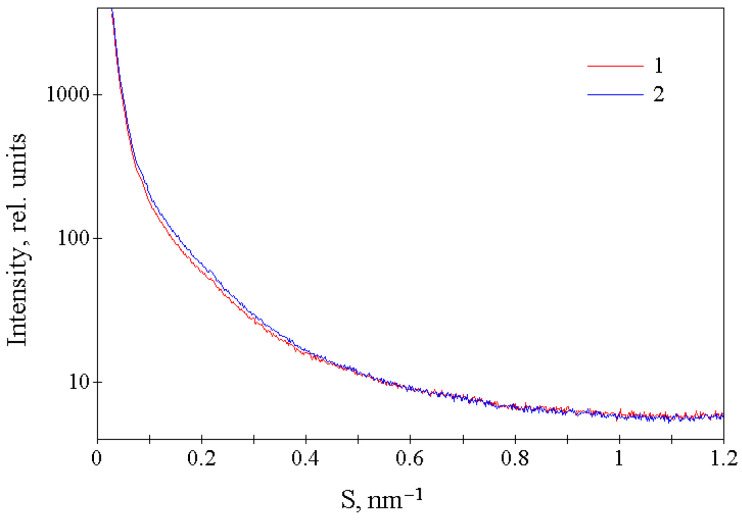
Intensity of small-angle X-ray scattering by tire material crushed in an explosion-circulation plant (1—CR 0–1) and mechanically (2—RD 0.8).

The small-angle X-ray scattering curves for crumb rubber samples obtained using both types of technologies are also almost identical (Figure 6). There are no diffraction maxima observed on these curves, and the decrease in intensity in the initial sections of these curves (at 0.025 nm^−1^ < S < 0.075 nm^−1^) is described by a function I(S) = kS^−α^ with a value of α ≈ 3.9, which indicates the absence of such maxima up to values of S ≈ 0.025 nm^−1^. From small-angle X-ray scattering data, it follows that the structure of rubber crumbs obtained using both types of technologies does not have spatial order on a distance scale of ≈1–40 nm.

Thus, X-ray diffraction studies of crumb rubber samples obtained in an explosive-circulation plant and after mechanical treatment showed that they have a very similar X-ray amorphous structure without supramolecular ordering on a distance scale of ≈1–40 nm.

#### 3.2.3. Surface Topography

**Figure 7:**
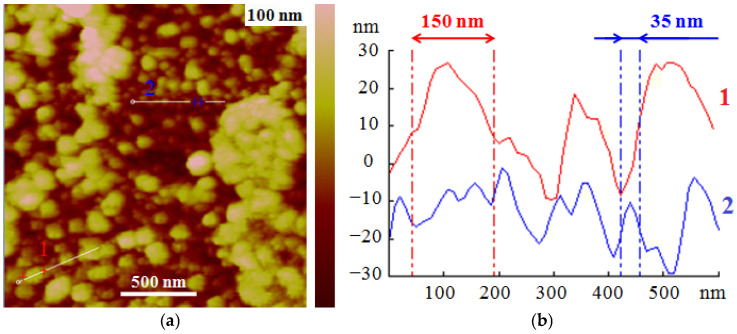
The surface topography of the crumb fraction. (**a**) Surface of the CR 3–5. (**b**) Sections along line 1 and 2 in the scan.

The surface topology of any fillers affects the quality of materials made from these fillers, so the surface topology of rubber crumb was studied using atomic force microscopy (AFM). When studying topography using the atomic force microscopy (AFM) method, it was not possible to obtain a scan size of more than 2 × 2 μm, since the samples were irregular in shape and their surface had high macroscopic heterogeneity. In images of various fractions (Figure 7), globular formations in a 10–50 nm range were observed. Their sizes were determined both by direct measurements in images and using section profiles (lines in the images). Globular formations were combined into clusters of various shapes. The cluster sizes varied in a wide range of 100–500 nm. The microscopic structure of the crumb rubber surface was approximately the same for all fractions considered. Nanoglobules, as well as small and large clusters of nanoglobules, randomly covered the entire surface of the studied regenerate samples. In the Figure 7, Sections 1 and 2 demonstrate the sizes of the clusters and globules, respectively.

The result is surprising because the tires used are an amorphous 3D mesh material filled with carbon black, processing additives, and oil. The listed fillers are not structural elements of the original rubber of worn tires and cannot provide such an image of the surface of the reclaimed crumbs. That is, globules cannot be formed directly from a mass of three-dimensional amorphous rubber during its destruction. Such a visual result could result, for example, from the destruction of expanded polystyrene, which is a continuous heterogeneous material consisting of individual foamed polymer macrospheres. Therefore, this feature of the crumb rubber surface should arise as a result of a specific process of explosive-circulation regeneration in worn tires. Probably, nanoglobules are secondary products that appear on the surface of the regenerate crumb as a result of some chemical-physical processes. Indeed, it was reported that even during the mechanical destruction of polyolefins, both saturated and unsaturated hydrocarbons containing C=C and even C≡C bonds were found in the reaction products [60]. Subsequently, compounds of higher molar mass can be formed from these unsaturated hydrocarbons due to the occurrence of radical processes. To confirm this assumption, a study was carried out on the composition of the surface and subsurface layers of fresh regenerated rubber crumb using various physical-chemical methods.

#### 3.2.4. Gel Permeation Chromatography

Since the surface of rubber crumb is presumably composed of high-molecular and low-molecular compounds that form nanoglobules, it was first necessary to determine the presence of high-molecular compounds on the surfaces. For this purpose, the extraction of these compounds from the surfaces of the regenerated rubber crumb was carried out (1 h at temperatures of 20 °C and 61 °C). Extraction temperature had no effect on the number of extracted substances. When these compounds were removed from the surface of crumb rubber with chloroform in a Soxhlet extractor, the mass loss was no more than 0.45%. The amount of extract correlated with the fractional composition of the crumbs. Smaller crumbs yielded more extractable substances. Since the number of extracted substances depended on the specific surface area of the rubber crumb, it was logical to assume that it was the substances that formed the nanoglobules observed using an atomic force microscope that were extracted.

**Figure 8:**
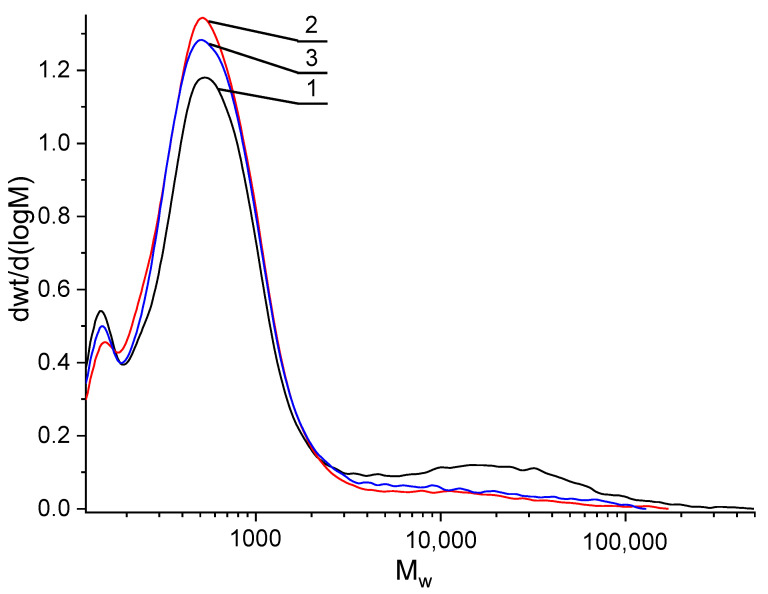
Molar mass distribution curves of extracts obtained at 20 °C for various fractions of crumb rubber: 1—CR 0–1; 2—CR 3–5; 3—CR 1–3.

The analysis of the extract by GPC showed (Figure 8) that it contains three fractions of substances with different molar masses. The lowest molar mass fraction can be classified as individual compounds, for example, monomers and dimers. In all samples of crumb rubber extract, the proportion of this fraction was about 10%. The next fraction is oligomeric, which is a complex mixture of unsaturated hydrocarbons with a molar mass of up to 700 daltons ($\bar{\mathrm{Mm}}=710$, $\bar{\mathrm{Mm}}/\bar{\mathrm{Mn}}=1.5$). The share of this fraction in the extract was the largest and amounted to more than 70%. This result is in good agreement with the results of thermal desorption mass spectrometry, as will be shown below. It was also possible to extract a relatively high-molecular fraction from rubber crumbs. In all samples of extract from crumb rubber, the proportion of this fraction did not exceed 10%. The molar mass of this fraction in samples CR 1–3 and CR 3–5 was $\bar{\mathrm{Mm}}=19,000$, $\bar{\mathrm{Mm}}/\bar{\mathrm{Mn}}=1.7$, and in sample RC 0–1—$\bar{\mathrm{Mm}}=27,000$, $\bar{\mathrm{Mm}}/\bar{\mathrm{Mn}}=2.0$.

#### 3.2.5. Gas Phase Analysis by Means of Gas Chromatography

To clarify the molecular composition of the rubber crumb surface (the presence of low-molecular compounds), additional analyses were carried out using gas chromatography and mass spectrometry.

Gas chromatographic analysis was used to analyze the gas phase of reclaimed rubber crumb fractions. Eighteen low-molar-mass compounds were detected in the molar mass range of 70–98 Da. Taking into account the value of the expected retention index (483–753 range), 11 compounds belonging to different classes of organic compounds were identified: 1-pentene (483), 2-methylbutene (494), n-pentane (500), 4-methyl-2-pentene (561), 2-methyl-1-pentene (583), hexane 600), methylhexene (667), methylhexane (671), 1,4-cyclohexadiene (699), methylcyclohexane (719), and methylbenzene (753) (the corresponding estimated retention index is given in parentheses for each compound). The identified components (C_2_–C_7_) could be formed as a result of the explosive-circulation destruction of both the rubber and the oil filling it.

#### 3.2.6. Mass Spectrometry

**Figure 9:**
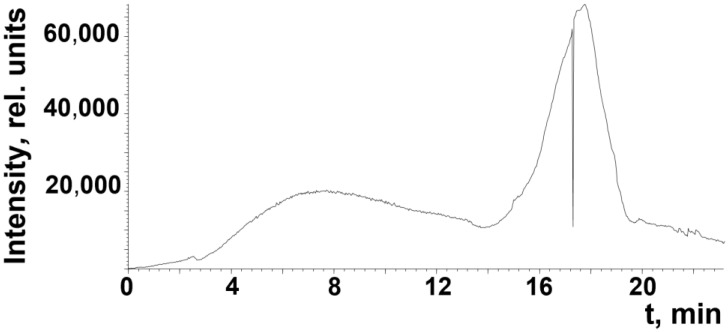
Total ion current curve recorded for the CR 0–1 rubber sample in the temperature range of 30–400 °C.

The samples of crumb rubber were studied by thermal desorption mass spectrometry. The mass spectra contained in the resulting total ion current curves were deciphered using the known patterns of fragmentation of organic compounds during electron ionization. An example of a thermal desorption curve for a sample of crumb rubber CR 0–1 is shown in Figure 9.

**Figure 10:**
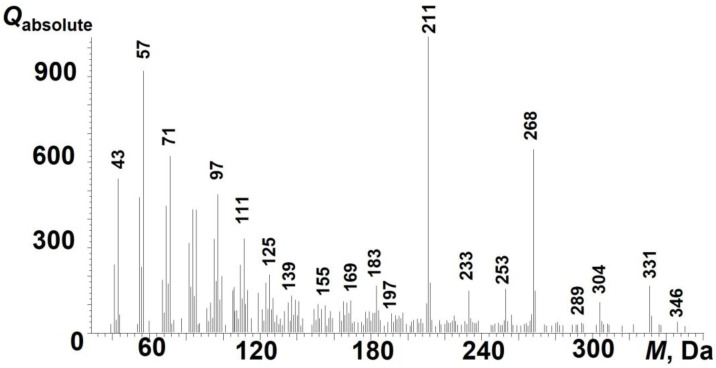
Mass spectrum of substances present in the first thermal desorption peak at 150 °C.

On the thermal desorption curve of the total ion current curve, there are two main peaks in the release of volatile products in the temperature ranges of 100–300 and 330–400 °C. A detailed examination of the mass spectra suggests that the destruction products contain saturated and unsaturated hydrocarbons, fatty acids, and sulfur-containing compounds. If in the first thermal desorption peak it is possible to identify some individual compounds, then in the second one there are complex products of the destruction of rubber and its components in the mass range of 100–500 Da. It can be argued that among the desorption products there are products of pyrolysis and mechanically activated destruction, which include unsaturated compounds. An example of characteristic ions of unsaturated compounds is given in Figure 10.

#### 3.2.7. X-Ray Photoelectron Spectroscopy

**Figure 11:**
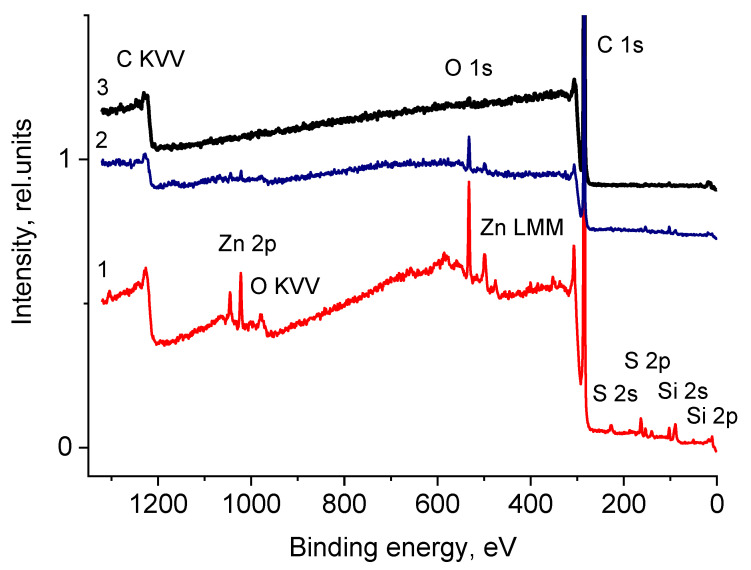
Survey spectra of the rubber crumbs: 1—CR 0–1; 2—CR 1–3; 3—CR 3–5.

Macromolecules of rubber used in tire manufacturing do not have polar groups. Therefore, rubber crumb obtained by mechanical grinding was not wetted. On the other hand, rubber crumb obtained by explosive-circulation technology had good wettability. This can only be explained by the occurrence of polar groups on the crumb surface; therefore, it was necessary to obtain results on the elemental and chemical composition of the near-surface region, determining its physical and chemical properties. To determine them, the surface-sensitive XPS method with an information depth of about 10 nm is widely used. Figure 11 shows the XPS C 1s spectra of samples CR 0–1, CR 1–3, and CR 3–5, and Table 1 contains the corresponding data on their elemental compositions.

**Figure 12:**
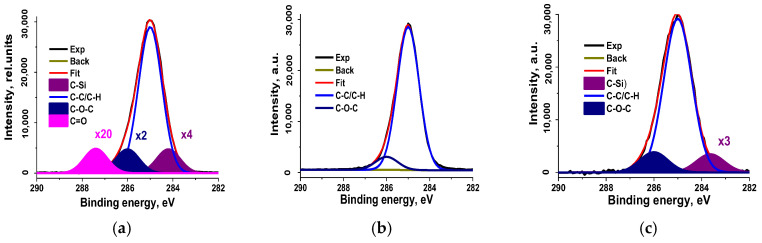
The C 1s high-resolution spectra of the rubber crumbs (**a**)—CR 0–1; (**b**)—CR 1–3; (**c**)—CR 3–5.

From these data, it follows that the samples differ both in the elements present in them and in their concentrations. Special attention was given to the analysis of carbon atoms in crumb rubber, including the examination of their chemical state through photoelectron and Auger spectra recordings. The C 1s spectra fitted with some Gaussian profiles are presented in Figure 12, and their characteristics are listed in Table 2.

The presence of C-O-C and C=O groups is naturally explained by the interaction of oxygen with radicals and C=C bonds present in the initial material. As mentioned above, such bonds are formed as a result of the explosive-circulation grinding of rubber according to the mechanism of mechanical destruction [56].

**Figure 13:**
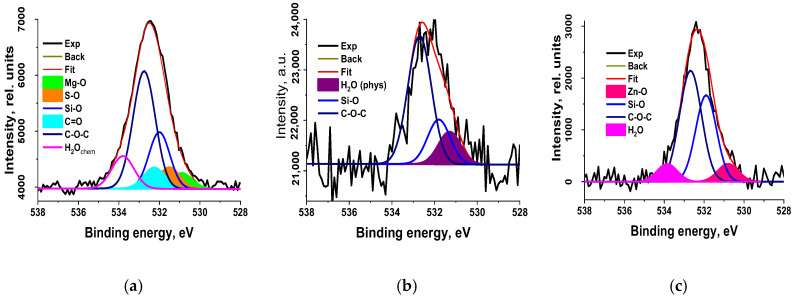
The O 1s high-resolution spectra of the rubber crumbs: (**a**)—CR 0–1; (**b**)—CR 1–3; (**c**)—CR 3–5.

The presence of polar groups on the crumb surface explains the high hydrophilicity mentioned above. It should be noted that the C KVV Auger spectra of the studied samples are close to the spectra of both polyethylene and polypropylene, which indicates a small effect of oxygen on carbon atoms in the uppermost layers, since the sampling depth of C KVV Auger electrons (3.6 nm) is approximately three times lower than that of the C 1s photoelectrons (10 nm). No signs of sp^2^-hybridization were detected in the C KVV spectra. However, the corresponding O 1s spectra show signals of chemisorbed and physisorbed water (Figure 13), despite the fact that the samples were in ultra-high vacuum, which may be due to the porosity of the material.

**Figure 14:**
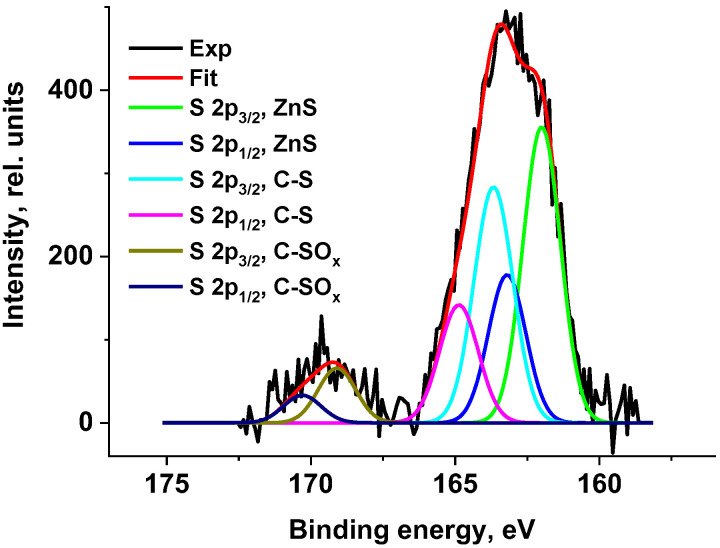
The S 2p high-resolution spectrum of the rubber crumbs CR 0–1.

Large amounts of sulfur are introduced into rubber mixtures as a vulcanizing agent. Therefore, three states at 162.7, 164.5, and 169.5 eV were detected in the photoelectron spectra for the Zn-S, C-S, and C-SO_x_ bonds, respectively (Figure 14). The concentration of sulfur in the form of C-S groups is comparable to that of oxygen. The appearance of sulfur in the form of SO_x_ groups is a consequence of the oxidation of sulfur atoms as a result of the explosive-circulation process.

**Figure 15:**
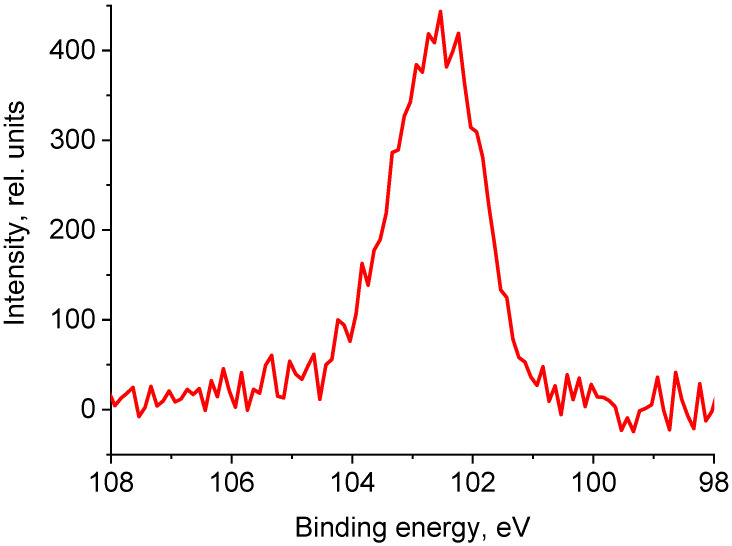
The Si 2p high-resolution spectrum of the rubber crumbs CR 0–1.

The presence of siloxane O-Si(O)-O groups, or SiO_2_, follows from the binding energy of the Si 2p peaks (~102.6 eV) and the fitting of the C 1s photoelectron spectra. In addition, C-Si bonds are observed in fairly high concentrations (Figure 15). The detection of peaks belonging to heteroatoms of zinc, silicon, and magnesium in the spectra can be attributed to various factors. One of them is the use of various technological additives in non-vulcanized rubber mixtures necessary for the production of tires [61,62]. Zn-O and Zn-S bonds were identified in the O 1s and S 2p photoelectron spectra (Figure 13 and Figure 14). Mg was recorded in an oxidized state (E_b_(Mg 1s) = 1304.7 eV).

Thus, the data on the chemical composition of the surface obtained by XPS explain the appearance of the wettability of rubber crumbs with water as a result of the appearance of a large number of polar, hydrophilic, primarily oxygen-containing groups.

## 4. Conclusions

The most popular product of waste tire recycling is rubber crumb, which is used as a filler in bitumen, rubberized cement–concrete mixtures, polymer concretes based on epoxy resin, and polymer composites. Good compatibility between rubber crumb and the matrix is a necessary condition for ensuring the high quality of such compositions. This compatibility is achieved through surface modification of the crumb by methods such as devulcanization, twin-screw extrusion, chemical modification, and plasma treatment. However, any modification represents an additional technological process that requires both time and financial resources.

This article provides, for the first time, a detailed description of a new patented technological process for regenerating worn tires using explosion-circulation technology. It also presents the results of a study on the physical and chemical properties of the resulting rubber crumb.

Good wettability with water was observed in almost all fractions of the rubber crumb regenerate, which can be attributed solely to the presence of polar groups on the crumb surface. However, the fraction with the smallest crumb size (0–1 mm) exhibited poor wettability due to the presence of a significant amount of non-wettable textile cord fibers. The excellent wettability of the explosion-circulation crumb eliminates the need for an additional technological process to modify its surface.

The modification of road-building bitumen with rubber crumb led to a significant improvement in its operational properties. Stiffness decreased from 217 to 161 MPa at −16 °C, while the complex shear modulus measured in the rheometer increased from 416.100 to 580.300 Pa at 300 °C.

X-ray diffraction studies of the regenerate, conducted in the region of large scattering angles and small-angle X-ray scattering, revealed that the regenerate possesses an X-ray amorphous structure without supramolecular ordering on a distance scale of approximately 1–40 nm.

Atomic force microscopy (AFM) enabled the detection of globular formations, ranging in size from 10 to 50 nm, on the surface of the amorphous rubber crumb. These formations were combined into clusters of various shapes, with cluster sizes varying widely between 100 and 500 nm.

Using gas chromatography, low-molecular-weight compounds in the mass range of 70–98 Da were detected. These compounds, which are easily desorbed from the surface of the rubber crumb, belong to various classes of organic compounds, including alkanes, alkenes, cycloalkanes, and cycloalkenes.

Some individual compounds, such as 2-phenylbenzothiazole and 2-thiolbenzothiazole, along with degradation products in the mass range of 100–500 Da, were identified using the thermal desorption mass spectrometric method. These degradation products are complex byproducts resulting from the destruction of rubber and its components.

The analysis of the chloroform extract of the crumb using gel chromatography revealed three fractions of substances with distinct molecular weights, as indicated by the molecular weight distribution curves: a low-molecular-weight fraction in the range of 100–200 Da (≈10%), an oligomeric fraction with a mass of up to 700 Da (more than 70%), and a relatively high-molecular-weight fraction with $\bar{\mathrm{Mw}}=$ 18,000–30,000 (less than 10%).

In the X-ray photoelectron spectroscopy (XPS) spectra of the crumb, signals with binding energies corresponding to polar groups such as C-O-C, C=O, Zn-O, Zn-S, C-S, C-SO_x_, O-Si(O)-O, and SiO_2_ were recorded. The presence of oxygen-containing groups can be attributed to the interaction of oxygen with radicals and C=C bonds present in the tire-destruction products.

Thus, the results from the conducted chemical and physicochemical studies allowed us to explain the good wettability of rubber crumb through the following processes. During the tire destruction process, in addition to rubber crumb, various low-molecular-weight compounds are formed, including both saturated and unsaturated compounds. These compounds are oxidized by oxygen present in the air within the explosion-circulation reactor. As a result of the condensation of these oxidation products on the surface of the rubber crumb, nanoglobules are formed. These nanoglobules consist of organic compounds with varying molecular weights and contain polar groups.

## 5. Patents

Nabok, A.A., RF Patent 2057014, 1996 [53].

Nabok, A.A., Zakharov, A.S., Patent WO 2012/053923 A1, 2012 [54].

## Figures and Tables

**Table 1:** XPS quantification data (at. %).

Sample	C	O	Mg	Si	Zn	S
CR 0–1	91.5	5.2	0.3	1.1	0.5	1.4
CR 1–3	99.0	0.8		0.2		
CR 3–5	94.4	3.8		1.5	0.3	

**Table 2:** Parameters of components in C 1s photoelectron spectra of rubber crumbs: E_b_—binding energy, W—peak width, and I_rel_—relative intensity.

Sample		C-Si	C-C/C-H	C-O-C	C=O;
CR 0–1	Eb	284.2	285.0	286.0	287.4
	W	1.03	1.03	1.03	1.03
	Irel	0.07	0.80	0.13	0.01
CR 1–3	Eb		285.0	286.0	
	W		1.02	1.03	
	Irel		0.92	0.08	
CR 3–5	Eb	284.2	285.0	286.0	
	W	1.17	1.17	1.17	
	Irel	0.05	0.84	0.11	

## Data Availability

The original contributions presented in this study are included in the article. Further inquiries can be directed to the corresponding author.

## Abbreviations

The following abbreviations are used in this manuscript:

CR	Crumb rubber
GPC	Gel permeation chromatography
AFM	Atomic force microscopy
XPS	X-ray photoelectron spectroscopy
SPME	Solid-phase microextraction
EC	Explosive charge
